# Dietary Fiber Intake Is Associated with HbA1c Level among Prevalent Patients with Type 2 Diabetes in Pudong New Area of Shanghai, China

**DOI:** 10.1371/journal.pone.0046552

**Published:** 2012-10-16

**Authors:** Junyi Jiang, Hua Qiu, Genming Zhao, Yi Zhou, Zhijie Zhang, Hong Zhang, Qingwu Jiang, Qiao Sun, Hongyan Wu, Liming Yang, Xiaonan Ruan, Wang-Hong Xu

**Affiliations:** 1 Key Laboratory of Public Health Safety, Department of Epidemiology, School of Public Health, Ministry of Education, Fudan University, Shanghai, People's Republic of China; 2 Pudong New Area Centers for Disease Control and Prevention, Shanghai, People's Republic of China; Fundación para la Prevención y el Control de las Enfermedades Crónicas No Transmisibles en América Latina (FunPRECAL), Argentina

## Abstract

**Background:**

Dietary factors play an important role in glycemic control in diabetic patients. However, little is known about their effects among Chinese diabetic patients, whose diets are typically abundant in fiber and high in glycemic index (GI) values.

**Methodology/Principal Findings:**

934 patients with type 2 diabetes and 918 healthy volunteers from Pudong New Area, Shanghai, China, were interviewed during the period of Oct-Dec, 2006 to elicit demographic characteristics and lifestyle factors. Dietary habits were assessed using a validated food frequency questionnaire. Anthropometric measurements, bio-specimen collection and biochemical assays were conducted at the interview according to a standard protocol. In this population, diabetic patients consumed lower levels of energy and macronutrients but had higher levels of fasting plasma glucose (FPG), glycolated hemoglobin A1c (HbA1c), triglyceride and body mass index than healthy adults. While the average consumption levels of the nutrients among diabetic patients did not vary along duration of the disease, the average levels of FPG and HbA1c increased with increasing duration. Regardless of diabetes duration, HbA1c level was observed lower in patients having a higher fiber or lower GI intake. Compared with those with the lowest tertile intake of fiber, the adjusted odds ratios (ORs) for poor glycemic control reduced from 0.75 (95%CI: 0.54–1.06) to 0.51 (95%CI: 0.34–0.75) with increasing tertile intake (*P* for trend <0.001).

**Conclusions:**

Dietary fiber may play an important role in reducing HbA1c level. Increasing fiber intake may be an effective approach to improve glycemic control among Chinese diabetic patients.

## Introduction

Type 2 diabetes is an important risk factor for micro-vascular and macro-vascular complications. Effective control of hyperglycemia, dyslipidemia and hypertension, either by medication or by lifestyle intervention, is crucial to decrease the incidence of stroke, myocardial infarction and renal disease, as well as the related premature death [1], [2]. Intervention studies have examined the impact of dietary intake on glycemic control, and found that higher intake of dietary fiber [3], [4] and lower intakes of dietary fat [5], glycemic index (GI) [6] and carbohydrate [7] improved glycemic control status. In the Nurses' Health Study, He, *et al* [8] observed a potential benefit of whole grain, cereal fiber and bran intake in reducing mortality and cardiovascular risk in diabetic patients. None of these studies, however, were conducted in Chinese population.

Chinese people consume more abundant types of foods, and have higher levels of dietary fiber and GI intake comparing with their western counterparts [9]. It is reported that average level of total and soluble fiber intake in Chinese diabetic patients were 26.5 and 10.4 gram per day (g/d), respectively [10], above the moderate amount of fiber intake recommended by the American Diabetes Association (ADA) (total, 24 g/d; 8 g/d of soluble fiber and 16 g/d of insoluble fiber) [11]. However, the status of glycemic control and prevalence of complications in diabetic patients in China have been not satisfactory [12].

To evaluate the association of dietary factors with diabetic control status among Chinese patients with type 2 diabetes, we conducted a cross-sectional study including 934 adult patients from Pudong New Area of Shanghai, China. Our results may help to better understand the role of dietary factors in the control of type 2 diabetes.

## Materials and Methods

### Study participants

In this cross-sectional design, a total of 979 adults diagnosed with type 2 diabetes were randomly selected from the Diabetes Administration Rosters in communities of Shanggang, Zhoujiadu, Huamu, Puxin, Weifang, Jinyang, Meiyuan and Jichang of Pudong New Area of Shanghai in Oct, 2006. All patients were diagnosed with type 2 diabetes by doctors according to ADA criteria: 1) fasting plasma glucose ≥7.0 mmol/L; or 2) two-hour plasma glucose ≥11.1 mmol/L during an oral glucose tolerance test; 75-g glucose load should be used; or 3) a random plasma glucose concentration ≥11.1 mmol/L in persons with symptoms of hyperglycemia or hyperglycemic crisis. Exclusion criteria included the occurrence of a cardiovascular event during the previous 6 months, advanced congestive heart failure, unstable angina, major depression and dementia. Of the 934 patients interviewed, 41.7% were male. The mean age of the participants was 64.5 (SD, 10.1) years old.

At the same time, a total of 918 adult volunteers free of diabetes were recruited from the spouses and neighbors of the diabetic patients. The mean age of these volunteers was 57.7 (SD, 9.9) years old, and 291(31.7%) were male.

The study was approved by Fudan University Institutional Review Board (IRB00002408, FWA00002399). Written informed consent was obtained from each participant before data collection.

### Data collection

A structured in-person interview was conducted for each subject by trained interviewers to collect information on demographic characteristics, duration of diabetes, age at onset of diabetes, diagnosis of hypertension, presence of dyslipidemia, use of tobacco and alcohol. Presence of hypertension, dyslipidemia and coronary heart disease were defined by a positive response to the question of “Have you ever been diagnosed with hypertension/dyslipidemia/coronary heart disease by a doctor?". Family history of diabetes was defined as positive if any first- or second-degree relative had type 2 diabetes. Smoking was defined as at least 1 cigarette per day for at least 6 months, and alcohol use was defined as drinking alcohol at least 3 times a week for more than 6 months.

Dietary habit was assessed using an interviewer-administered food frequency questionnaire (FFQ) modified based on a validated FFQ [13]. The FFQ specifies 103 food items, covering 90% of food items commonly consumed in Shanghai. For each food item, participants were asked to report how frequently (daily, weekly, monthly, annually or never) and how long (months per year) they consumed the food, followed by a question on the amount of consumption in *liang* (1 *liang* = 50 g) per unit of time in previous 12 months. For liquid foods such as milk, juice and beverage, the amount of intake was reported in milliliter (ml) and was transformed into gram in the analysis. The daily intakes of oil, salt and sugar were calculated as the average level consumed by each family member of the participant.

Nutrient content from the Chinese Food Composition Tables was applied to estimate nutrient intake from all food items and groups, and to obtain GI values for most food items [14]. For the remaining food items, we referenced Foster-Powell *et al*'s report to obtain their GI values [15]. Glycemic load (GL) from each food was calculated by multiplying the food's GI value by the carbohydrate content of the food and the average amount of the food consumed per day. Total dietary GL was then produced by summing these products over all food items. Dietary GI was derived by dividing the dietary GL by the amount of carbohydrate intake, thus yielding a weighted average GI for each individual's diet. We excluded from the analysis 3 women who had total energy intake <500 kcal/d or >3500 kcal/d and 5 men with energy intake of <800 kcal/d or >4000 kcal/d.

### Metabolic phenotype measurements

At the interview, each participant was measured for his/her body height, weight, waist circumference, hip circumference, systolic blood pressure (SBP), and diastolic blood pressure (DBP) according to a uniform and standardized protocol. Body mass index (BMI) was calculated as weight (kg) divided by height squared (m^2^).

After at least 10 hours of overnight fasting, a 1∼1.5 ml venous blood specimen was collected in a vacuum tube containing sodium fluoride for the measurement of plasma glucose and HbA1c, and a 3∼3.5 ml non-anticoagulated venous blood specimen was collected simultaneously for the measurement of total cholesterol (TC), triglyceride (TG), high density lipoprotein cholesterol (HDLC) and low density lipoprotein cholesterol (LDLC).

Enzymology methods were used to measure the fasting plasma glucose (FPG) level (GOD-PAP), concentrations of TG (GPO-PAP) and TC (CHOD-PAP) on an Automatic Biochemical Analyzer (HITACHI 7170A, Hitachi, Ltd, Tokyo, Japan). Levels of HDLC and LDLC were measured using a selective inhibition method. HbA1c was tested using ion exchange chromatography on DS5 Glycated Hemoglobin Analyzer (DREW DS5, Drew Scientific Co. Ltd, Cumbria, UK). Quality control of the assays was assessed internally and externally. The inter-assay coefficient of variation was <1.82% for FPG (SD<0.23 mmol/L), <1.38% for TG (SD<0.02 mmol/L), <1.54% for TC (SD<0.08 mmol/L), <1.6% for HDLC (SD<0.01 mmol/L), <5.3% for LDLC (SD<0.21 mmol/L), and 6.13% for HbA1c (SD<0.77).

### Statistical analysis

Statistical analyses were conducted utilizing SAS statistical software 9.2 (SAS Institute Inc., Cary, NC). Differences on demographic factors by diabetic status or glycemic control status were evaluated using χ^2^ test for categorical variables or non-parameter Wilcoxon test for continuous variables. Partial correlation analysis was conducted to evaluate the linear correlations of HbA1c levels with amounts of dietary intake. A generalized linear regression model was applied to compare the average levels of biochemical measurements and average levels of dietary intake. An unconditional logistic regression model was employed to estimate the adjusted odds ratios (ORs) and 95% confident intervals (CIs) of dietary intake with glycemic control status among diabetic patients. Natural log transformation was applied to normalize the distribution of biochemical measurements before parametric methods were used in data analysis. All statistical tests were based on two-sided probability.

## Results

Compared with the recruited healthy volunteers, diabetic patients were older and less educated, had more family history of diabetes and higher prevalence of hypertension, dyslipidemia and coronary heart disease (CHD), as shown in Table 1. Among 934 prevalent diabetic patients, 488 (52.3%) had an HbA1c level of ≥7.0%. These patients, compared with those having an HbA1c level of <7.0%, appeared to have less education, younger age at diagnosis of diabetes, longer duration of diabetes, lower prevalence of hypertension and were more likely to use oral hypoglycemia drug and insulin. No significant difference was observed between the two groups with regard to age, sex, smoking, alcohol consumption, family history of diabetes, presence of dyslipidemia and prior history of CHD.

**Table 1:** Comparison of demographic characteristics of participants of the study.

Characteristics	Healthy adults (N = 918)	Diabetic patients (N = 934)	*P value*	Diabetic patients		*P value* a
				HbA1c<7.0% (N = 446)	HbA1c≥7.0% (N = 488)	
Age, years (mean, SD)	57.7 (9.9)	64.5 (10.1)	*<0.001*	64.6 (10.1)	64.4 (10.1)	*0.825*
Sex, male, n (%)	291 (31.7)	389 (41.7)	*<0.001*	191 (42.8)	198 (40.6)	*0.487*
Educational level, high school or below, n (%)	816 (88.9)	864 (92.5)	*0.008*	400 (89.9)	465 (94.9)	*0.004*
Family history of diabetes, yes, n (%)	88 (9.6)	298 (31.9)	*<0.001*	130 (29.2)	168 (34.4)	*0.084*
Diagnosis age of DM, years (mean, SD)	–	55.2 (10.4)	–	56.4 (10.4)	54.2 (10.0)	*0.002*
Duration of DM, years (mean, SD)	–	9.2 (6.4)	–	8.2 (6.3)	10.2 (6.3)	*<0.001*
Prior history of hypertension, n (%)	293 (31.9)	518 (55.5)	*<0.001*	265 (59.4)	253 (51.8)	*0.020*
Prior history of dyslipidemia, n (%)	61 (6.6)	97 (10.4)	*0.004*	55 (12.3)	42 (8.6)	*0.062*
Prevalence of CHD, n (%)	46 (5.0)	132 (14.1)	*<0.001*	73 (16.4)	59 (12.1)	*0.061*
Current smoking, n (%)						
Men	134 (46.1)	152 (39.1)	*0.068*	70 (36.7)	82 (41.4)	*0.336*
Women	6 (1.0)	5 (0.9)	*0.944*	2 (0.8)	3 (1.0)	*1.000*
Current alcohol consumption, n (%)						
Men	113 (33.8)	108 (27.8)	*0.002*	49 (25.7)	59 (29.8)	*0.362*
Women	21 (3.4)	10 (1.8)	*0.108*	5 (2.0)	5 (1.7)	*1.000*
Oral hypoglycemic drug use, n (%)	–	762 (81.5)	–	341(76.5)	421(87.0)	*<0.001*
Insulin use, n (%)	–	88 (9.4)	–	31(7.0)	57(11.8)	*0.012*

Missing values (1 for age, education, diagnosis age of DM, duration of DM in diabetic patients; 1 for current alcohol consumption in healthy adults; 4 for oral hypoglycemic drug use and insulin use) were excluded;

a χ^2^ test for categorical variables or non parameter Wilcoxon test for continuous variables.

As shown in Table 2, the diabetic patients, both men and women, seemed to remain a stable dietary habit along duration of diabetes (*P*>0.05 for all tests). These patients, either with a short or a long duration of type 2 diabetes, had lower levels of energy, carbohydrate, protein, and fat intake than did healthy volunteers after adjusting for age and other potential confounders (*P*<0.01). Dietary fiber (soluble only), GI and GL intake were also lower in diabetic patients than in healthy volunteers, but the difference reached significance for GI and GL only among women.

**Table 2:** Average levels of dietary intake by diabetic status and duration of type 2 diabetes.

Dietary factors^a^ (Mean, SD)	Healthy adults (N = 918)	Diabetic patients (N = 934)	*P value* b	Diabetic patients, duration of DM, years			*P value* c
				<5 (N = 230)	5–9 (N = 316)	≥10 (N = 387)	
Men							
Energy, kcal/d	1931.2 (1.4)	1644.1 (1.3)	*<0.001*	1740.8 (1.3)	1600.0 (1.3)	1615.5 (1.3)	*0.225*
Carbohydrate, g/d	323.6 (1.4)	268.2 (1.3)	*0.003*	282.5 (1.3)	263.9 (1.3)	261.6 (1.3)	*0.583*
Protein, g/d	68.9 (1.4)	61.5 (1.5)	*0.003*	65.1 (1.5)	58.3 (1.4)	62.0 (1.4)	*0.467*
Fiber, g/d	10.2 (1.6)	9.2 (1.7)	*0.232*	9.9 (1.6)	8.4 (1.6)	9.6 (1.7)	*0.045*
Fat, g/d	40.9 (1.6)	37.1 (1.6)	*0.002*	40.0 (1.5)	35.3 (1.6)	36.9 (1.6)	*0.485*
Average GI	61.5 (1.1)	60.4 (1.2)	*0.323*	61.0 (1.2)	61.4 (1.1)	59.2 (1.2)	*0.230*
Average GL	104.7 (1.4)	87.6 (1.4)	*0.347*	93.3 (1.4)	84.0 (1.4)	86.5 (1.4)	*0.670*
Women							
Energy, kcal/d	1695.2 (1.3)	1410.5 (1.3)	*<0.001*	1445.2 (1.4)	1438.3 (1.4)	1367.4 (1.3)	*0.438*
Carbohydrate, g/d	279.2 (1.3)	226.3 (1.4)	*<0.001*	234.0 (1.4)	231.4 (1.4)	217.9 (1.4)	*0.878*
Protein, g/d	61.8 (1.4)	52.9 (1.5)	*<0.001*	54.3 (1.5)	53.7 (1.5)	51.2 (1.5)	*0.973*
Fiber, g/d	10.2 (1.6)	8.5 (1.7)	*0.460*	8.8 (1.7)	8.4 (1.7)	8.3 (1.7)	*0.868*
Fat, g/d	38.4 (1.6)	33.4 (1.6)	*<0.001*	33.5 (1.6)	33.8 (1.7)	32.9 (1.5)	*0.797*
Average GI	59.9 (1.1)	59.2 (1.2)	*<0.001*	59.3 (1.1)	59.6 (1.2)	58.7 (1.2)	*0.733*
Average GL	92.1 (1.4)	74.2 (1.4)	*<0.001*	76.8 (1.4)	75.7 (1.4)	71.5 (1.4)	*0.783*

a Continuous variables were all natural LOG transformed before entering models;

b Generalized linear model adjusting for age, BMI and energy intake;

c Generalized linear model adjusting for age, BMI, oral hypoglycemic drug use, insulin use and energy intake.

Presented in Table 3 was the comparison of average levels of metabolic indicators by diabetic status and by duration of type 2 diabetes. After adjustment of age and sex, higher levels of FPG, HbA1c, TG and BMI and a lower level of LDLC were observed among diabetic patients than in healthy adults. Among diabetic patients, the average levels of FPG, HbA1c increased and BMI decreased with increasing duration of the disease after adjusting for age, sex, oral hypoglycemic drug use and insulin use (*P* for trend <0.05). No significant upward trend was observed for average levels of TC, TG, LDLC and HDLC along with duration of the disease.

**Table 3:** Average levels of metabolic indicators by diabetic status and duration of type 2 diabetes.

Indicators^a^ (Mean, SD)	Healthy adults	Diabetic patients	*P value* b	Diabetic patients, duration of DM, years			*P value* c
	(N = 918)	(N = 934)		<5 (N = 230)	5–9 (N = 316)	>9 (N = 387)	
FPG, mmol/L	5.3 (1.2)	8.0 (1.4)	*<0.001*	7.2 (1.3)	8.1 (1.4)	8.5 (1.4)	*<0.001*
HbA1c (%)	5.9 (1.1)	7.4 (1.2)	*<0.001*	6.9 (1.2)	7.4 (1.2)	7.7 (1.2)	*<0.001*
TC, mmol/L	4.5 (1.2)	4.4 (1.2)	*0.003*	4.4 (1.2)	4.4 (1.2)	4.4 (1.2)	*0.733*
TG, mmol/L	1.3 (1.7)	1.4 (1.8)	*<0.001*	1.5 (1.8)	1.4 (1.7)	1.4 (1.8)	*0.508*
LDLC, mmol/L	2.8 (1.3)	2.7 (1.4)	*<0.001*	2.7 (1.4)	2.7 (1.3)	2.7 (1.4)	*0.177*
HDLC, mmol/L							
Men	1.1 (1.3)	1.1 (1.2)	*0.998*	1.1 (1.2)	1.1 (1.1)	1.2 (1.1)	*0.111*
Women	1.3 (1.3)	1.3 (1.3)	*0.224*	1.3 (1.3)	1.3 (1.2)	1.3 (1.3)	*0.266*
BMI	24.8 (1.1)	25.6 (1.1)	*0.005*	25.8 (1.2)	25.7 (1.1)	24.8 (1.1)	*0.002*

a Continuous variables were all natural LOG transformed before entering models;

b Generalized linear model adjusting for age and gender;

c Generalized linear model adjusting for age, gender, oral hypoglycemic drug use and insulin use.

**Figure 1:**
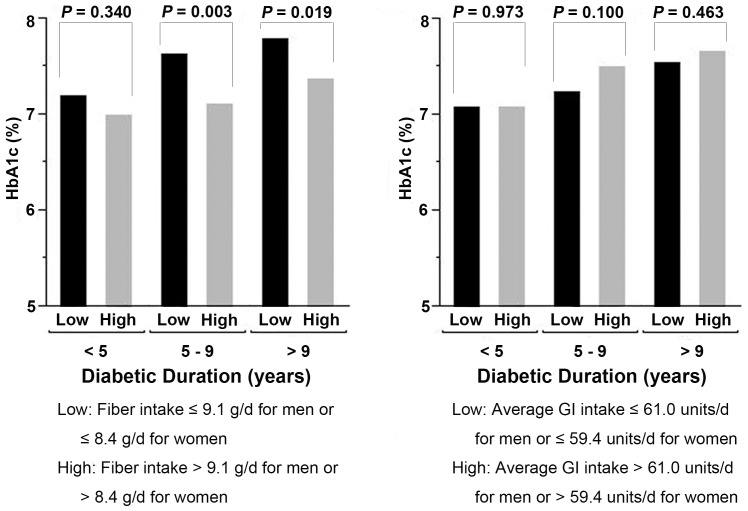
Average levels of HbA1c by duration of type 2 diabetes and dietary fiber or GI intake. The low and high fiber or GI intakes were classified by the medians of consumption in men and women, respectively. Means of HbA1c level were adjusted for age (as a continuous variable), sex (male/female), duration of type 2 diabetes (as a continuous variable), BMI (as a continuous variable), oral hypoglycemic drug use (yes/no), insulin use (yes/no) and energy intake (as a continuous variable).

In diabetic patients, HbA1c level was negatively correlated with dietary fiber intake (r = −0.079, p = 0.017), and positively with dietary GI intake (r = 0.070, p = 0.034) after adjusting for age, sex, oral hypoglycemic drug use and insulin use. No significant linear correlation was observed for HbA1c level with other nutrients (data not shown). Therefore, we further compared the average levels of HbA1c by dietary fiber and GI intake among diabetic patients (Figure 1). Regardless of duration of type 2 diabetes, HbA1c level was consistently higher in patients consuming lower level of fiber. HbA1c level was also higher among patients having higher GI intake, but the difference did not reach significance.

We further evaluated the associations of dietary factors with glycemic control status which was defined “poor" as HbA1c ≥7.0% (Table 4). Compared with the patients having the lowest tertile intake of dietary fiber, the adjusted ORs for poor glycemic control decreased from 0.75 (95%CI: 0.54–1.06) for those having medium intake to 0.51 (95%CI: 0.34–0.75) for those with the highest tertile intake (*P* for trend <0.001). No significant association was observed for other dietary factors.

**Table 4:** Associations of dietary intake with glycemic control status among diabetic patients.

Dietary intake^a^	HbA1c, % (Mean, SD)	Glycemic control status, N (%)		OR (95%CI)^b^	*P for trend*
		Controlled (HbA1c<7.0%)	Uncontrolled (HbA1c≥7.0%)		
Energy (kcal/d)					
Low	7.5 (1.4)	143 (32.2)	165 (34.2)	1.00	
Medium	7.6 (1.7)	139 (31.3)	170 (35.3)	1.03 (0.74, 1.44)	
High	7.5 (1.7)	162 (36.5)	147 (30.5)	0.93 (0.71, 1.22)	*0.300*
Carbohydrate (g/d)					
Low	7.6 (1.5)	137 (30.9)	171 (35.5)	1.00	
Medium	7.5 (1.6)	147 (33.1)	163 (33.8)	0.85 (0.59, 1.23)	
High	7.5 (1.8)	160 (36.0)	148 (30.7)	0.69 (0.40, 1.16)	*0.166*
Protein (g/d)					
Low	7.6 (1.5)	135 (30.4)	172 (35.7)	1.00	
Medium	7.5 (1.6)	151 (34.0)	159 (33.0)	0.75 (0.52, 1.08)	
High	7.5 (1.8)	158 (35.6)	151 (31.3)	0.62 (0.38, 1.01)	*0.052*
Fiber (g/d)					
Low	7.7 (1.5)	126 (28.4)	182 (37.8)	1.00	
Medium	7.5 (1.5)	148 (33.3)	162 (33.6)	0.75 (0.54, 1.06)	
High	7.4 (1.8)	170 (38.3)	138 (28.6)	0.51 (0.34, 0.75)	*<0.001*
Fat (g/d)					
Low	7.5 (1.5)	139 (31.3)	168 (34.9)	1.00	
Medium	7.4 (1.6)	167 (37.6)	144 (29.9)	0.72 (0.51, 1.02)	
High	7.7 (1.8)	138 (31.1)	170 (35.3)	1.16 (0.76, 1.77)	*0.630*
Average GI					
Low	7.5 (1.6)	145 (32.7)	163 (33.8)	1.00	
Medium	7.4 (1.7)	165 (37.2)	144 (29.9)	0.88 (0.64, 1.23)	
High	7.7 (1.6)	134 (30.2)	175 (36.3)	1.25 (0.90, 1.75)	*0.184*
Average GL					
Low	7.6 (1.6)	141 (31.8)	166 (34.4)	1.00	
Medium	7.6 (1.6)	139 (31.3)	172 (35.7)	1.00 (0.70, 1.45)	
High	7.5 (1.7)	164 (36.9)	144 (29.9)	0.68 (0.41, 1.12)	*0.154*

a Dietary factors were classified as low, medium and high intake by the tertiles in men and in women, respectively. The cut points for energy intake were 1444.06 and 1842.84 kcal/d in men and 1268.88 and 1623.31 kcal/d in women; for carbohydrate were 248.93 and 295.77 g/d in men and 205.85 and 267.65 g/d in women; for protein intake were 50.89 and 72.42 g/d in men and 44.88 and 62.71 g/d in women; for fiber intake were 7.12 and 11.32 g/d in men and 6.73 and 10.51 g/d in women; for fat were 31.02 and 44.62 g/d in men and 28.74 and 40.61 g/d in women; for average GI were 56.53 and 64.58 units/d in men and 55.88 and 62.95 units/d in women; for average GL were 65.57 and 99.67 units/d in men and 76.34 and 87.55units/d in women.

b OR, odds ratio; 95%CI, 95% confidence interval; OR: adjusted for age (as a continuous variable), sex (male/female), duration of type 2 diabetes (as a continuous variable), BMI (as a continuous variable), oral hypoglycemic drug use (yes/no), insulin use (yes/no) and energy intake (as a continuous variable).

## Discussion

In this cross-sectional study including 934 Chinese prevalent patients with type 2 diabetes from Pudong New Area of Shanghai, China, we observed a stable after-diagnosis dietary habit, upward trend of HbA1c level along with duration of diabetes, a lower average level of HbA1c related with higher fiber intake, and a probably protective effect of dietary fiber on glycemic control status. Our findings have several implications in improving diabetic control status and preventing complications among Chinese diabetic patients.

Firstly, in this study, the patients with an HbA1c level of ≥7.0% were diagnosed with type 2 diabetes 2.2 years earlier than those with a lower HbA1c level, and had a 2 years longer duration of the disease. The result is consistent with several previous studies, in which younger age at diagnosis and longer duration were regarded as independent predictors for poor glycemic control in diabetic patients [16]–[18]. The result suggests that more attention in glycemic control should be paid to the younger patients.

Secondly, the significant difference in dietary habit between patients and healthy adults suggests that a diabetic patient could greatly change his/her dietary habit just due to diagnosis of type 2 diabetes. The stable dietary habit along the duration of the disease in patients, on the other hand, indicates that a new dietary habit, once established, would remain unchanged for a long time. These results implicate the importance of the time point of diagnosis in establishing a new “good" dietary habit. Dietary intervention and health education should be extensively elicited at the time point.

In both healthy and diabetic subjects of this study, the average levels of dietary fiber intake reached moderate amount of fiber intake recommended by ADA. In this population with high average level of dietary fiber intake, dietary fiber was still linked to better glycemic control status, supporting the beneficial effect of dietary fiber on the control of type 2 diabetes. We also find that, it was GI, but not carbohydrate or GL intake, that was correlated with HbA1c level. The results indicate that the quality of the carbohydrates consumed may play a more important role than the quantity of the macronutrient in the control of type 2 diabetes. Dietary GI and GL are two physiological indexes of the metabolic effects of dietary carbohydrates [15], [19]. While GI is used to characterize foods that contain carbohydrates according to their postprandial blood glucose response and hence their effect on blood insulin levels [19], [20], GL is introduced to quantify the overall estimate of postprandial glycemia by combining the GI value and the quantity of carbohydrates consumed [15], [20]. Our finding of the improved glycemic control status related to dietary fiber intake is consistent with the previous studies [4], [6], [21]. The insignificant adverse effect of GI intake, on the other hand, was supported only by Wolever *et al*'s report [22], but not consistent with most previous studies in which a low-GI diet significantly improved glycemic control by reducing HbA1c levels among diabetic patients [6], [23]–[25]. It is of note that, even if it effected on glucose metabolism, a low-GI diet was not a practical intervention tool in controlling diabetes [26]. Increasing dietary fiber intake may be an effective approach.

Regarding the role of carbohydrate and GL intake, the results have been controversial. While Lau, *et al* [27] reported that the inverse association of carbohydrate and daily GL intake with HOMA-IR can be explained by dietary fiber, Esposito, *et al* [25] found that diet high in GL was significantly associated with higher HbA1c and postmeal glucose levels in a dose-dependent manner among 901 diabetic outpatients even after adjusting for dietary fiber intake. Whereas Haimoto, *et al* [7] found that a 6-month low carbohydrate diet (30% of energy intake from carbohydrate) decreased HbA1c level from 10.9% to 7.4% among 33 severe diabetic outpatients, Wolever, *et al* [22] did not observe a beneficial effect of a low-carbohydrate diet in glycemic status. The null associations of carbohydrate and GL intake with HbA1c level in our patients indicate that the two dietary factors play a limited role in glycemic control in our population.

In this study, we did not find an association of HbA1c levels with dietary protein intake, consistent with Larsen *et al*'s results [28], but not with Pearce *et al*'s findings [29]. The null association with dietary fat is also inconsistent with a randomized clinical trial, in which both a low-fat vegan diet and a traditional diet according to the ADA guidelines decreased HbA1c level in 99 individuals with type 2 diabetes after a 22-week follow up [5].

Finally, we observed that HbA1c level was consistently associated with dietary fiber intake regardless of duration of the disease, suggesting that dietary intervention may make a difference at any time during the progression of the disease.

The strengths of this study included the validated food frequency questionnaire, a standardized protocol for body measurements, and stringent quality control in lab assays. Due to the nature of the cross-sectional design, however, we could not elucidate the role of dietary factors in glycemic control. Moreover, the amount of energy intake appeared lower than those reported in previous studies [30], raising our concern on possible recall bias. However, the potential underestimation of dietary intake, if any, would result in a non-differential misclassification bias, which may have biased our results towards the null.

In summary, this small-scale cross-sectional study indicates the potential role of dietary fiber in glycemic control. Our results, if confirmed, may have clinic and public health implications in diabetic control among Chinese adults.
